# Expression and regulation of ATL9, an E3 ubiquitin ligase involved in plant defense

**DOI:** 10.1371/journal.pone.0188458

**Published:** 2017-11-21

**Authors:** Fengyan Deng, Tingwei Guo, Mitchell Lefebvre, Steven Scaglione, Christopher J. Antico, Tao Jing, Xin Yang, Weixing Shan, Katrina M. Ramonell

**Affiliations:** 1 Department of Biological Sciences, University of Alabama, Tuscaloosa, AL, United States of America; 2 College of Life Sciences, Northwest A&F University, Yangling, Shaanxi, China; 3 Statistics Research and Consulting Lab, Culverhouse College of Commerce and Business Administration, University of Alabama, Tuscaloosa, AL, United States of America; 4 College of Plant Protection, Northwest A&F University, Yangling, Shaanxi, China; Iwate University, JAPAN

## Abstract

Plants are continually exposed to a variety of pathogenic organisms, including bacteria, fungi and viruses. In response to these assaults, plants have developed various defense pathways to protect themselves from pathogen invasion. An understanding of the expression and regulation of genes involved in defense signaling is essential to controlling plant disease. ATL9, an *Arabidopsis* RING zinc finger protein, is an E3 ubiquitin ligase that is induced by chitin and involved in basal resistance to the biotrophic fungal pathogen, *Golovinomyces cichoracearum* (*G*. *cichoracearum*). To better understand the expression and regulation of *ATL9*, we studied its expression pattern and the functions of its different protein domains. Using p^ATL9^:*GUS* transgenic *Arabidopsis* lines we found that *ATL9* is expressed in numerous tissues at various developmental stages and that GUS activity was induced rapidly upon wounding. Using a GFP control protein, we showed that ATL9 is a short-lived protein within plant cells and it is degraded via the ubiquitin-proteasome pathway. ATL9 contains two transmembrane domains (TM), a RING zinc-finger domain, and a PEST domain. Using a series of deletion mutants, we found that the PEST domain and the RING domain have effects on ATL9 degradation. Further infection assays with *G*. *cichoracearum* showed that both the RING domain and the TM domains are important for ATL9’s resistance phenotype. Interestingly, the PEST domain was also shown to be significant for resistance to fungal pathogens. This study demonstrates that the PEST domain is directly coupled to plant defense regulation and the importance of protein degradation in plant immunity.

## Introduction

Plants have developed precise inducible defense systems to respond to potential pathogens. Inducible plant defenses that are initiated through pathogen recognition fall into two major categories [1]. The first type is triggered by the recognition of Pathogen-Associated Molecular Patterns (PAMPs) by plant pattern-recognition receptors (PRRs). This class of resistance is referred as PAMP Triggered Immunity (PTI) [2]. Well-characterized PAMPs of plant pathogens include bacterial flagellin, elongation factor Tu (EF-Tu), and the fungal-associated elicitor chitin. The second type of immunity, Effector Triggered Immunity (ETI), is activated upon perception of a pathogen Avirulence (Avr) protein by a plant resistance (R) protein. This type of resistance is often characterized by a hypersensitive cell death response (HR) at the infection site [3]. Although knowledge of how plant defense systems are activated is increasing rapidly, our understanding about resistance attenuation and termination is still limited.

Emerging evidence suggests that E3 ligases play critical roles in the plant response to variety of stimuli [4, 5]. The RING E3 Ligase KEEP ON GOING (KEG), which was found as a negative regulator of abscisic acid (ABA) signaling [6,7], was found to be an important factor in plant jasmonate (JA) signaling [8]. Recently, Copeland et al. found that the evolutionarily conserved *Arabidopsis* E3 ligase, AtCHIP, is involved in positive regulation of plant disease resistance at low temperature [9].

Several different families of E3 ubiquitin-ligases that are involved in different steps of plant immune responses have been classified based on their structural features and mechanism of action [10, 11, 12, 13]. The ATL family, a particular family of RING finger E3 ligases, includes at least 80 members in *A*. *thaliana* and 121 in *O*. *sativa*. Genes of this family are characterized by their rapid induction after elicitor treatment, their structure that includes a highly conserved RING-H2 zinc-finger domain, and at least one transmembrane domain (TM) [14]. The *Arabidopsis ATL2* gene is specifically induced by chitin and may function in the early steps of an elicitor-response pathway and in both the SA- and JA-mediated defense-response pathway [15, 16]. Noda et al. showed that *ATL54* plays an important role in both secondary cell wall biosynthesis and in programmed cell death [17]. Two other *Arabidopsis* ATL family members, *ATL6* and *ATL31*, are involved in the defense response to the bacterial pathogen *Pseudomonas syringae* pv. *tomato* DC3000 [18]. In a previous study, our group found that *Arabidopsis* ATL9 has E3 ubiquitin ligase activity and is involved in chitin- and NADPH oxidase-mediated defense responses [19].

*ATL9*, also known as *ATL2G* (At2g35000), is a member of the *ATL* gene family. The ATL9 protein is unique among the ATL family for that it contains a PEST domain. PEST domains are associated with proteins that have a short half-life and it is suggested that it acts as a signal peptide for protein degradation [20, 21]. Although no evidence has been reported showing that the PEST domain is involved in pathogen resistance, there are other genes containing a PEST domain that are known to be involved in plant immunity, such as the tomato gene, *Ve* [22] and the *Arabidopsis* flagellin receptor gene *FLS2* [23].

In this work, we have expanded on our previous studies of ATL9, focusing on its expression and regulation, as well as analyzing the roles of its protein domains in plant immunity. We present evidence concerning the spatial and temporal expression of *ATL9* using P^ATL9^:*GUS* transgenic *Arabidopsis* plants. Furthermore, we examined the post-translational regulation of *ATL9* using GFP or GUS reporters in onion epidermal cells, tobacco epidermal cells, and transgenic *Arabidopsis*. Additionally, we show that the localization of ATL9, its E3 ligase activity, and its PEST domain are important to its resistant phenotype.

## Materials and methods

### Biological materials

*Arabidopsis thaliana* ecotype Columbia (Col-0) was used as a control in all experiments. T-DNA insertional mutants of *ATL9* (*At2G35000*, CS24736, SALK_066755) were obtained from the *Arabidopsis* Biological Resource Center (ABRC, Ohio State University). To identify homozygous T-DNA mutants, PCR reactions were performed using the following primers: LP 5'-CAATTCTTGTAAGAGGCATGG-3', RP 5'-TCCTAAACCAACAAGGTGACG-3' and T-DNA primer: 5'-TAGCATCTGAATTTCATAACCAATCTCGATACAC-3'. Plants were grown under controlled conditions in a growth chamber at 22°C day/19°C night with 16 hrs of light per 24 hrs and 50% RH.

The fungal pathogen used in this work was *Golovinomyces cichoracearum UCSC1*. *G*. *cichoracearum* stock culture was grown on cucumber and maintained at 22°C day/19°C night with 16 hrs of light per 24 hrs and 85% relative humidity.

### Disease assessments

Powdery mildew inoculations and disease assessments were carried out as previously described [24]. In brief, *Arabidopsis* seeds were planted in soil and grown in a growth chamber. After four weeks, plants were inoculated with powdery mildew and placed in a growth chamber under the same temperature and light conditions except at 85% relative humidity. Disease development was assessed in a qualitative manner by tracking the appearance of powdery symptoms on inoculated leaves over a period of 10 days post inoculation (dpi). Leaves at 5 dpi were harvested and stained in a trypan blue solution (25 mg/ml trypan blue in a 1:1:1 solution of water, glycerol, and lactic acid) overnight and then decolorized in 95% ethanol overnight. The number of conidiophores per colony was determined from at least 18 leaves taken from 18 plants per genotype. Statistical significance among samples was analyzed using both ANOVA and post hoc tests.

### Promoter *GUS* transgenic *Arabidopsis* construct and assay

A 1325 bp promoter region of *ATL9* and a 2462 bp P^ATL9^:*ATL9* region were amplified from *Arabidopsis* genomic DNA using primers containing an attB recombination site. Primer sequences are as follows:

5’-GGGGACAAGTTTGTACAAAAAAGCAGGCTTCTAAATTACAAAATGACCCACG-3’ (F);

5’-GGGGACCACTTTGTACAAGAAAGCTGGGTCTTGAAGATCATCGTATGGAAA-3’ (R for P^ATL9^)

5’-GGGGACCACTTTGTACAAGAAAGCTGGGTCTTACACTCGTTCATCTGGTCGGAGC-3’ (RP for P^ATL9^:*ATL9*).

PCR products were recombined with pDONR221 (Invitrogen) using BP Clonase (Invitrogen). Correct clones were recombined with pMDC162 and pMDC163 respectively [25] using LR Clonase Enzyme Mix (Invitrogen). All plasmid inserts were sequenced prior to transformation and verified constructs were transformed into *Agrobacterium tumefaciens* strain GV3010 and subsequently transformed into wild-type Col-0 via the floral-dip method [26]. Transformants were screened on 1/2 MS media containing 50 μg/mL hygromycin B. Resistant plants were then transferred to soil and allowed to set seed. Homozygous transgenic lines selected from T3 lines were subjected to histochemical staining.

Seedlings and tissues were stained overnight at 37°C in GUS staining buffer (containing 0.5M sodium phosphate buffer (pH 7.2), 10% Triton X-100, 100mM potassium ferrocyanide, 100mM potassium ferricyanide and 10mM X-Gluc) [27]. Samples were destained for up to 8 hours in 95% Ethanol and observed. Wound treatment was carried out by scratching 40-day old transgenic plant leaves with tweezers. The leaves were stained immediately after wound treatment. Chitin treatment was carried out by bathing the 40-day old transgenic plant leaves in 1 mg/mL CSC (chitin) for 24 hours before staining. Control transgenic *Arabidopsis* were bathed in water.

### Complementation assay

All *ATL9* sequences were PCR amplified from Col-0 genomic DNA using the Iproof High Fidelity Polymerase (Bio-Rad). Two-step fusion PCR was used for P^ATL9^:*ATL9ΔTM*, P^ATL9^:*ATL9ΔPEST and* P^ATL9^:*ATL9ΔRING* amplification using the following primers:

First step:

P^ATL9^:*ATL9ΔTM*:

5’-GGGGACAAGTTTGTACAAAAAAGCAGGCTTCTAAATTACAAAATGACCCACG-3’ (P^ATL9^:*ATL9* 1F)

5’-ACTGTGCGTTGCTTCGACGCATTTGAAGATCATCGTATGGA-3’ (*ATL9ΔTM* 1R)

5’-ATGCGTCGAAGCAACGCACAGT-3’ (*ATL9ΔTM* 2F)

5’-GGGGACCACTTTGTACAAGAAAGCTGGGTCTTACACTCGTTCATCTGGTCGGAGC-3’ (*ATL9*R)

P^ATL9^:*ATL9ΔPEST*:

P^ATL9^:*ATL9*F

5’-GTCGTCGTCGTCACCTTGTTG-3’ (*ATL9ΔPEST 1*R)

5’-ACAAGGTGACGACGACGACAGAGGAATGGTTTTGGAATCT-3’ (*ATL9ΔPEST* 2F)

*ATL9*R

P^ATL9^:*ATL9ΔRING*:

5’-GTGAAACACGTAGCAACAAGG-3’ (*ATL9ΔRING* 1R);

5’-CCTTGTTGCTACGTGTTTCAC-3’ (*ATL9ΔRING* 2F);

*ATL9*R Second step:

P^ATL9^:*ATL9ΔTM*:

*ATL9ΔTM* 1F & *ATL9ΔTM* 2R

P^ATL9^:*ATL9ΔPEST*:

*ATL9ΔPEST* 1F & *ATL9ΔPEST* 2R

P^ATL9^:*ATL9ΔRING*:

*ATL9ΔRING* 1F & *ATL9ΔRING* 2R

The PCR product was recombined with pDONR221 (Invitrogen) using BP Clonase (Invitrogen). Verified clones (including P^ATL9^:*ATL9*) were recombined with pMDC99 [25] using LR Clonase Enzyme Mix (Invitrogen). 35S:*ATL9ΔTM*, 35S:*ATL9ΔPEST and* 35S:*ATL9ΔRING* were constructed in the same method except that 5’ forward primer was 5’-GGGGACAAGTTTGTACAAAAAAGCAGGCTTCATGGCGATCCTCGACACAAAG-3’ (*ATL9* 1F) and the destination vector was pMDC32. Correct clones were then transformed into *A*. *tumefaciens* strain GV3010 and subsequently transformed into *atl9* T-DNA insertional mutant CS24736 via the floral-dip method [26]. Transformed plants were screened on 1/2 MS media containing 50μg/mL hygromycin B. Resistant plants were then transferred to soil and allowed to set seed. Homozygous transgenic lines were selected from T3 lines for disease assessments.

### Analysis of protein degradation in onion epidermal cells and recombinant DNA procedures

Recombinant DNA (35S:*ATL9*-*GFP*, 35S:*ATL9ΔTM*-*GFP*, 35S:*ATL9ΔPEST*-*GFP*, 35S:*ATL9ΔRING*-*GFP*) were constructed in the same way as 35S:*ATL9*, 35S:*ATL9ΔTM*, 35S:*ATL9ΔPEST and* 35S:*ATL9ΔRING*, except that the destination vector was pMDC83. The endoplasmic reticulum luminal marker fused to mCherry, ER-rk, was acquired from the ABRC (stock number CD3-959). The recombinant *ATL9-GFP* constructs and ER-rk were co-bombarded into the fresh onion epidermal cells by using Tungsten microcarriers and PDS-1000/He Particle Delivery System (Bio-Rad). Bombardment was executed following the manufacturer’s protocol and then the onion epidermal cells were incubated on 1/2 MS media in the dark at room temperature (22–23°C) for 12 hours before observing the expression.

The protein synthesis inhibitor cycloheximide (CHX) was added 12 hours post bombardment to stop protein synthesis. Thereafter, GFP and mCherry fluorophores were visualized at different time points post CHX treatment (0 h, 6 h) using a Nikon Eclipse 90i epifluorescent microscope (Nikon, Melville, NY) equipped with an OptiGrid imaging system (Qioptiq, Paris, France) with FITC HYQ (Excitation: 460–500 nm; Emission: 510–560 nm) and TRITC HYQ (Excitation: 530–560 nm; Emission: 590–650 nm) filters. NIS-Elements software (Version 3.2, Nikon) was used to generated and/or merged images.

### Protein immunoblotting and half-life analysis in tobacco leaves

Recombinant plasmids (35S:*ATL9-GFP*, 35S:*ATL9ΔPEST*-*GFP*, 35S:*ATL9ΔRING*-*GFP*) were transformed into *A*. *tumefaciens* strain GV3010. *Agrobacterium* cultures were grown overnight at 28°C, until the *Agrobacterium* density reached an OD_600_ = 1.0. 1 mL of the culture was pelleted, washed and re-suspended in 1 mL of infiltration media. The suspended *A*.*tumefaciens* cells were then infiltrated into 5-week old *Nicotiana benthamiana* leaves. GFP was monitored 48 hours post infiltration using fluorescent microsocopy (Nikon, Melville, NY). Leaves with GFP were cut into 1 mm^2^ pieces and transferred to plates with 1/2 MS media containing 0.5 mg/ml CHX under vacuum. Small tobacco leaf pieces were collected for protein extraction at different time points (0 h and 6 h) and processed for quantitative protein immunoblotting using anti-GFP polycolonal antibody (Invitrogen).

## Results

### Expression profile of *ATL9*

**Fig 1:**
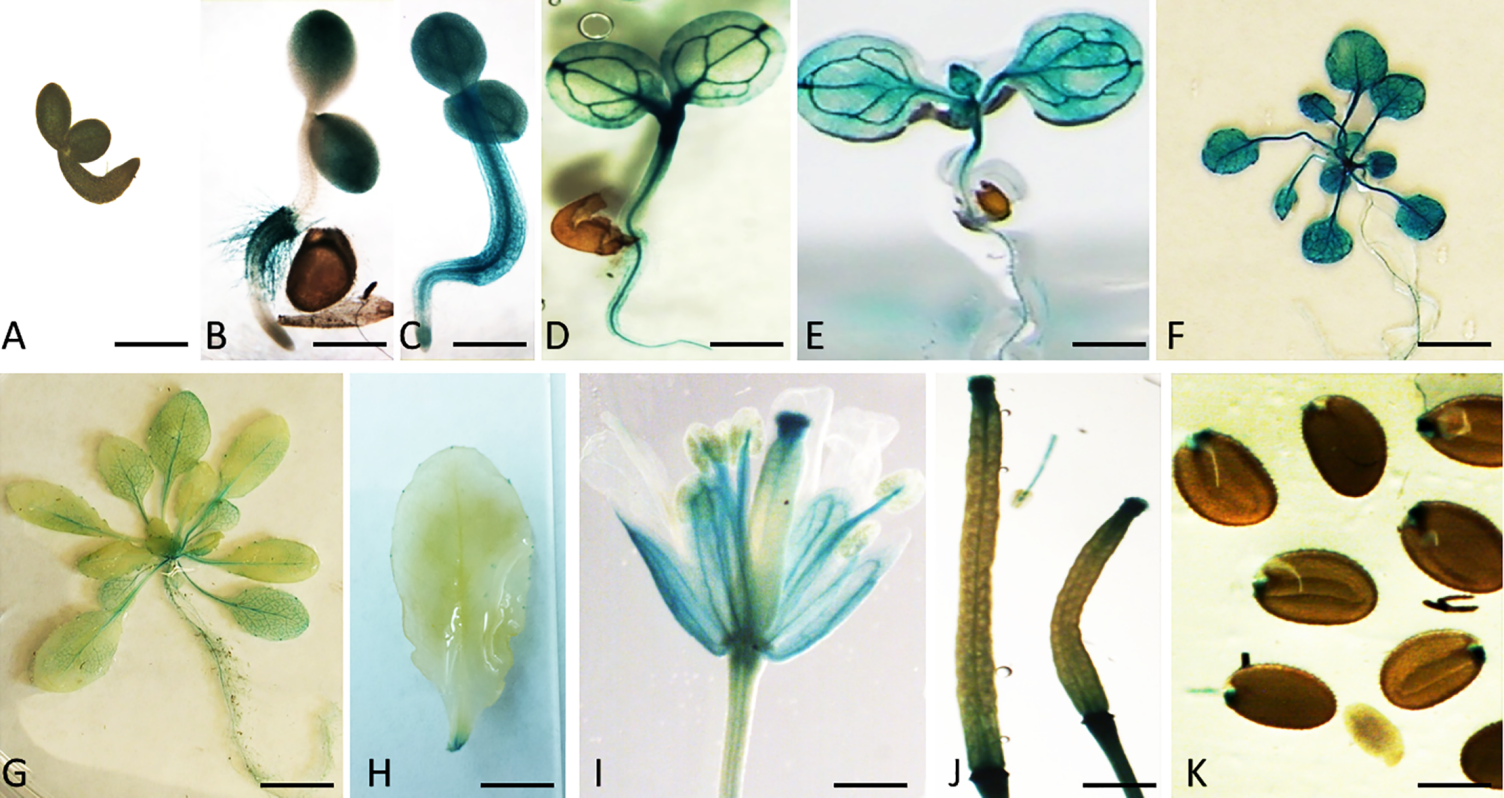
Expression of *ATL9* in *Arabidopsis* tissues at different developmental stages. Different developmental stages of P^ATL9^:*GUS* were subjected to GUS staining at 37°C for at least 4 hours. (A) 2-day old germinating seedling. (B) 3-day old seedling. (C) 4-day old seedling. (D) 7-day old seedling. (E) 2-week old seedling. (F) 3-week old plant. (G) 4-week old plant. (H) 5-week old leaf. (I) Flower. (J) Siliques. (K) Seeds. Seedlings and tissues were stained overnight at 37°C in GUS staining buffer. Samples were destained for up to 8 hours in 95% Ethanol and observed. Scale bar: 0.5mm in A-E; 1mm in F; 2mm in G; 5mm in H; 0.5mm in I; 1mm in J; 0.2mm in K.

In order to study the spatial and temporal expression of *ATL9*, transgenic *Arabidopsis* plants were generated to express *GUS* under the control of a 1325 bp *ATL9* promoter region. Homozygous transgenic lines were selected from the T3 generation and subjected to GUS staining at 37°C for at least 4 hours. Little P^ATL9^:*GUS* activity was detected in 2-day old germinating seedlings (Fig 1A). P^ATL9^:*GUS* activity was detected in cotyledons, hypocotyls and root hairs in 3-day old seedlings (Fig 1B) and was observed in all tissues by day 4 (Fig 1C). As the seedlings grew, P^ATL9^:*GUS* activity changed from uniform expression in all tissues to expression predominantly in the vasculature and hypocotyl of 7-day old seedlings (Fig 1D) or in the vasculature, leaves, petioles and stems of 2-week and 3-week old seedlings (Fig 1E and 1F). In 4-week old transgenic plants P^ATL9^:*GUS* activity was still observed in the vasculature but expression was much weaker (Fig 1G). After the transition to flowering, other than the wounding area, P^ATL9^:*GUS* activity was no longer detected in the vascular tissue of leaves or petioles, as previously observed in younger plants (Fig 1H). However, significant GUS activity was now observed in the sepals, the anther filaments and stigma of mature flowers (Fig 1I), the tips and abscission zone of young developing siliques (Fig 1J), and at the micropylar end of the mature seeds (Fig 1K).

To demonstrate that GUS expression is not dependent on the developmental stage of *Arabidopsis*, but on *ATL9’s* expression regulation, we also monitored the GUS activity of another GUS transgenic line P^CRP1^:*GUS* (S1 Fig). Promoter region of CRP1 (Chloroplast RNA Processing 1) was cloned into destination vector with GUS reporter gene in the same way as P^ATL9^:*GUS*. No GUS activity was observed in 2-day old seedlings (S1A Fig). In addition, in all developmental stages, no GUS expression was detected in the roots or in the root hairs (S1A–S1D Fig). Similar to P^ATL9^:*GUS*, GUS activity was observed in the sepals and the anther filaments of mature flowers (S1E Fig). However, GUS activity in the anther filaments was only distributed in the vasculature, and no obvious GUS activity was detected in the stigma of flowers and siliques (S1E and S1F Fig), suggesting that ATL9 expression differs depending on the development stage and tissue Furthermore, upon wounding treatment, P^ATL9^:*GUS* activity in leaf tissue around the wound was rapidly induced (S2A Fig). Additionally, 4-week old P^ATL9^:*GUS* transgenic plants treated with chitin showed a significant increase in GUS activity in both leaves and petioles (S2B Fig). Taken together these results indicate that *ATL9* is regulated both temporally and spatially during *Arabidopsis* development.

### *ATL9* encodes a short-lived protein

**Fig 2:**
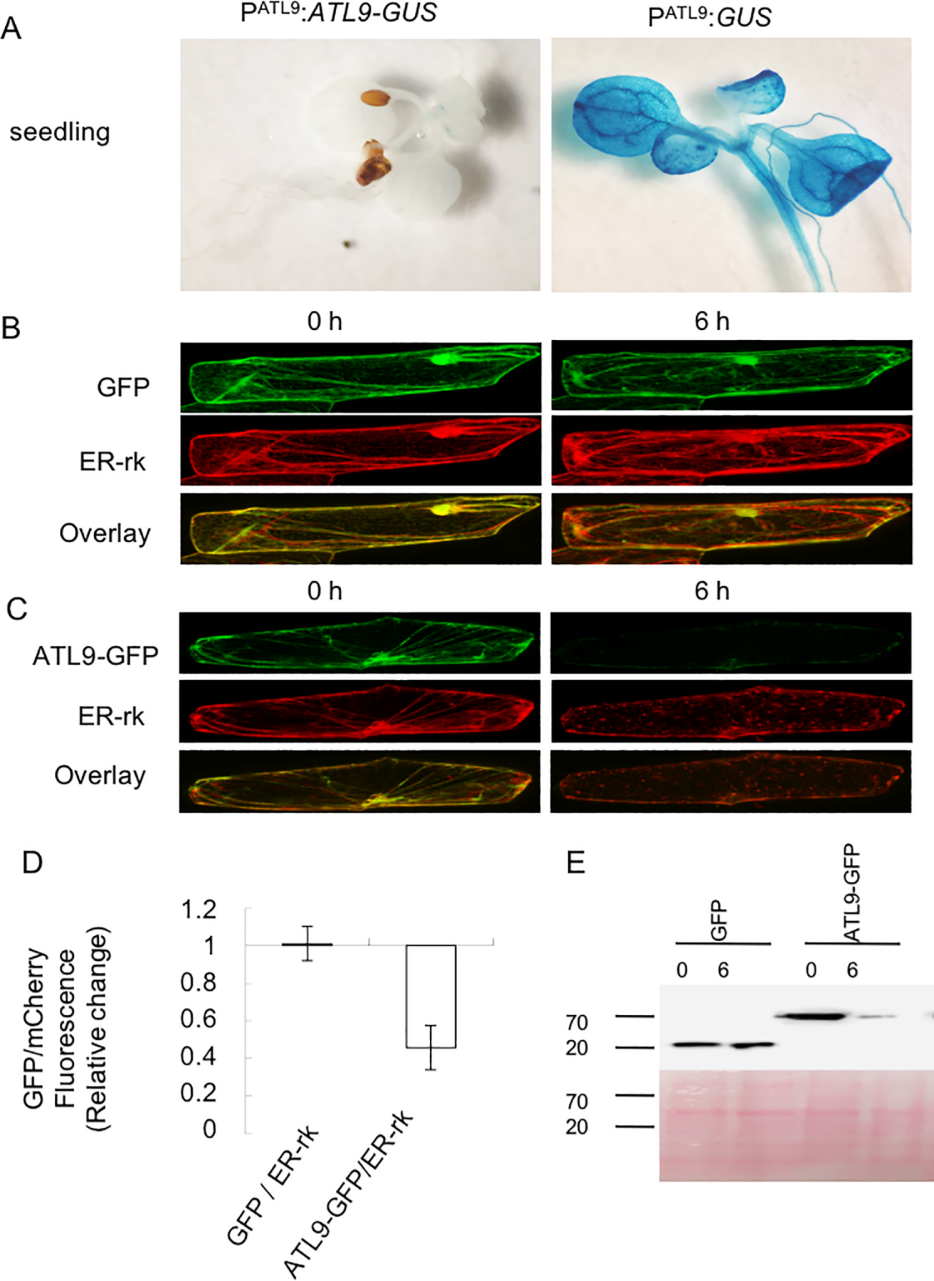
ATL9 is a short-lived protein. (A) GUS activity in P^ATL9^:*ATL9*-*GUS* and P^ATL9^:*GUS* transgenic *Arabidopsis*. (B, C) Degradation of free GFP (B) and ATL9-GFP (C) in onion epidermal cells. (D) Relative change of GFP/mCherry fluorescence intensity ratio of free GFP and ATL9-GFP. Onion epidermal cells were co-bombarded with ER-rk and either 35S:*ATL9*-*GFP* or 35S:*GFP*. CHX was added at 12 h after the bombardment to stop the protein synthesis. GFP/mCherry fluorescence intensity ratios were traced at different times after protein synthesis shut off (0 h, 6 h). (E) Measurement of GFP and ATL9-GFP’s half-life in tobacco leaves by western blot. The GFP and ATL9-GFP were transiently expressed in 5-week old *N*. *benthamiana* leaves. Then, total protein was extracted from leaves with observed GFP expression at different time points (0 h and 6 h) after CHX treatment. Western blots were performed using anti-GFP polyclonal antibody. Before blocking, the membrane was stained with Ponceau S solution in order to confirm equal amounts of protein loaded per lane.

Recent studies have shown that protein degradation is one of the most important events in regulating the plant innate immune response [28, 29]. Through the GUS staining experiment, we showed that GUS activity is almost invisible in seedlings and roots of P^ATL9^:*ATL9-GUS* transgenic plants, while is highly expressed in P^ATL9^:*GUS* transgenic plants (Fig 2A). We hypothesized that this difference could be related to post-translational regulation of *ATL9*. To better understand this, we utilized both onion epidermal cells and the tobacco transient expression system to study the post-translational regulation of *ATL9*. An N-terminal fusion of *ATL9* to *GFP* was constructed under the control of the CaMV 35S promoter (35S:*ATL9-GFP*). Inoculation results of 35S:*ATL9*-*GFP* complementary transgenic *Arabidopsis* showed that these plants were more resistant to the fungal pathogen than Col-0. In contrast, plants expressing only 35S:*GFP* were unable to rescue the defense phenotype of the *atl9* mutant. Taken together, these results indicate that ATL9-GFP is functional in our transgenic plants and *GFP* alone does not interrupt the function of ATL9 (S3 Fig).

The 35S:*ATL9-GFP* or 35S:*GFP* and a known endoplasmic reticulum (ER) luminal marker ER-rk (signal peptide of *AtWAK2*-*mCherry*-*HDEL*) were co-bombarded into onion epidermal cells [30]. GFP and mCherry fluorescence decay were monitored via confocal microscopy at different time points post protein translation shut off. Our results showed that free GFP and mCherry have long half-lives within the cell: 6 hours after protein translation termination, the GFP and mCherry fluorescence intensity remained significantly high (Fig 2B–2D). Compared to free GFP, the ATL9-GFP fusion protein has a shorter life span: at 6 hours post translation termination, the ATL9-GFP/mCherry ratio decreased significantly to 45.75% of the original ratio (ATL9-GFP/mCherry ratio at 0 hour post translation shut off) (Fig 2B–2D). These data suggest that ATL9 is a short-lived protein.

To further confirm that ATL9 is a short-lived protein, we utilized *N*. *benthamiana* to obtain protein for protein immunoblotting. Both ATL9-GFP and GFP alone were transiently expressed in 5-week old *N*. *benthamiana* leaves. Total protein was then extracted from leaves and GFP expression was observed at different time points (0 h and 6 h) after CHX treatment. Western blot results showed that ATL9-GFP accumulation was significantly decreased from 0 hour to 6 hour after protein expression termination (Fig 2E), while no noticeable change was observed in free GFP accumulation (Fig 2E). These data confirm that ATL9 has a shorter half-life compared to free GFP.

### *ATL9* is degraded by a proteasome-dependent mechanism

**Fig 3:**
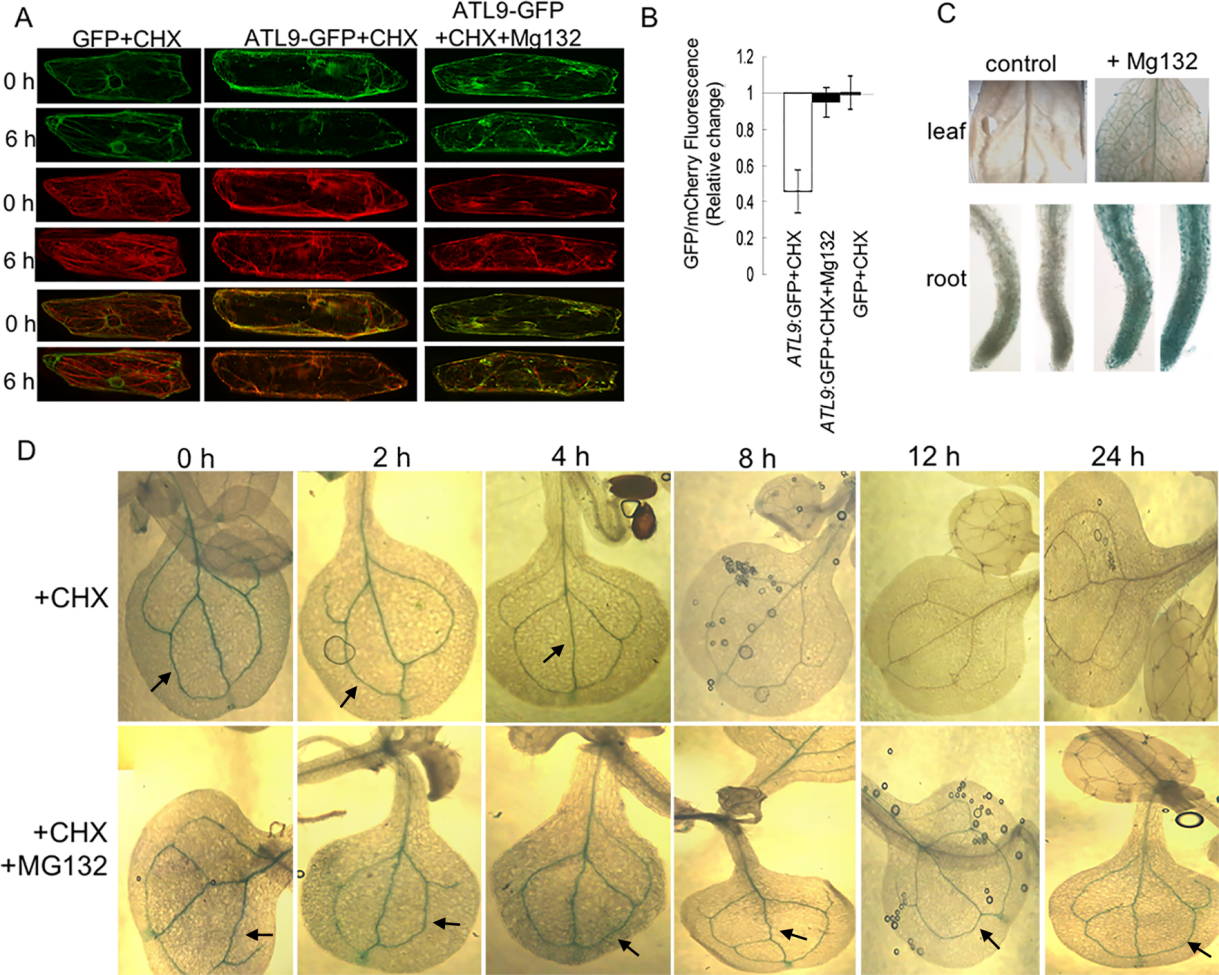
ATL9 is degraded by a proteasome-dependent mechanism. (A) Degradation of ATL9-GFP in onion epidermal cells. Methods used in this assay are as described in Fig 2 except that 100μM proteasome inhibitor MG132 was applied to the test samples at 12 h after bombardment to stop ubiquitin-proteasome mediated protein degradation. (B) Relative change of GFP/mCherry fluorescence intensity ratio of ATL9-GFP and free GFP. (C) GUS staining of P^ATL9^:*ATL9*-*GUS* transgenic plant leaves and roots. P^ATL9^:*ATL9-GUS* transgenic *Arabidopsis* seedlings were cultured in 1/2 MS media in the presence or absence of MG132 for 10 hours, and then subjected to GUS staining. (D) GUS staining of P^ATL9^:*ATL9*-*GUS* transgenic plants at different time points after CHX only or CHX and MG132 treatment. Two-week old P^ATL9^:*ATL9*-*GUS* transgenic *Arabidopsis* seedlings were cultured in 1/2 MS media with the presence of CHX alone or CHX and MG132, and then subjected to GUS staining at different time points.

The ubiquitin/26S proteasome system (UPS) constitutes the major protein degradation pathway in the cell. To determine whether proteasome function is required for ATL9 degradation, we monitored the GFP fluorescence decay of ATL9-GFP in onion epidermal cells at different time points after treatment with the proteasome inhibitor MG132 and the protein synthesis inhibitor CHX. Although treatment combined MG132 and CHX can cause stress on cells, especially in relation to the ER distribution, it doesn’t change ATL9-GFP localization when compared to ER-rk. Therefore, we used them to study ATL9 degradation. Our data showed that without MG132 treatment, fluorescent emission of ATL9-GFP decreased rapidly, compared to mCherry and GFP. Meanwhile, the GFP/mCherry ratio in ATL9-GFP/ER-rk expression cells is similar to that of GFP/ER-rk expression cells after the application of MG132 (Fig 3A and 3B). This suggests that protesome inhibitor MG132 can prevent ATL9 from degradation via a proteasome-dependent pathway.

To further confirm this result, P^ATL9^:*ATL9*-*GUS* transgenic *Arabidopsis* plants were utilized to trace ATL9 protein degradation. We found that, after 10 hours of MG132 application, the accumulation of ATL9-GUS protein was significantly increased in P^ATL9^:*ATL9-GUS* transgenic *Arabidopsis* plants (Fig 3C). Furthermore, we traced ATL9 protein degradation in P^ATL9^:*ATL9*-*GUS* transgenic *Arabidopsis* plants by monitoring GUS accumulation at different time points after CHX or CHX and MG132 treatment. We found that, after 4 hours of CHX only treatment, almost no ATL9-GUS accumulation was detected, while the ATL9-GUS level was similar between different time points after CHX and MG132 application (Fig 3D). Taken as a whole, these results suggest that ATL9 is degraded via a proteasome-dependent pathway.

### The PEST domain and the RING domain contribute to the short half-life of ATL9

**Fig 4:**
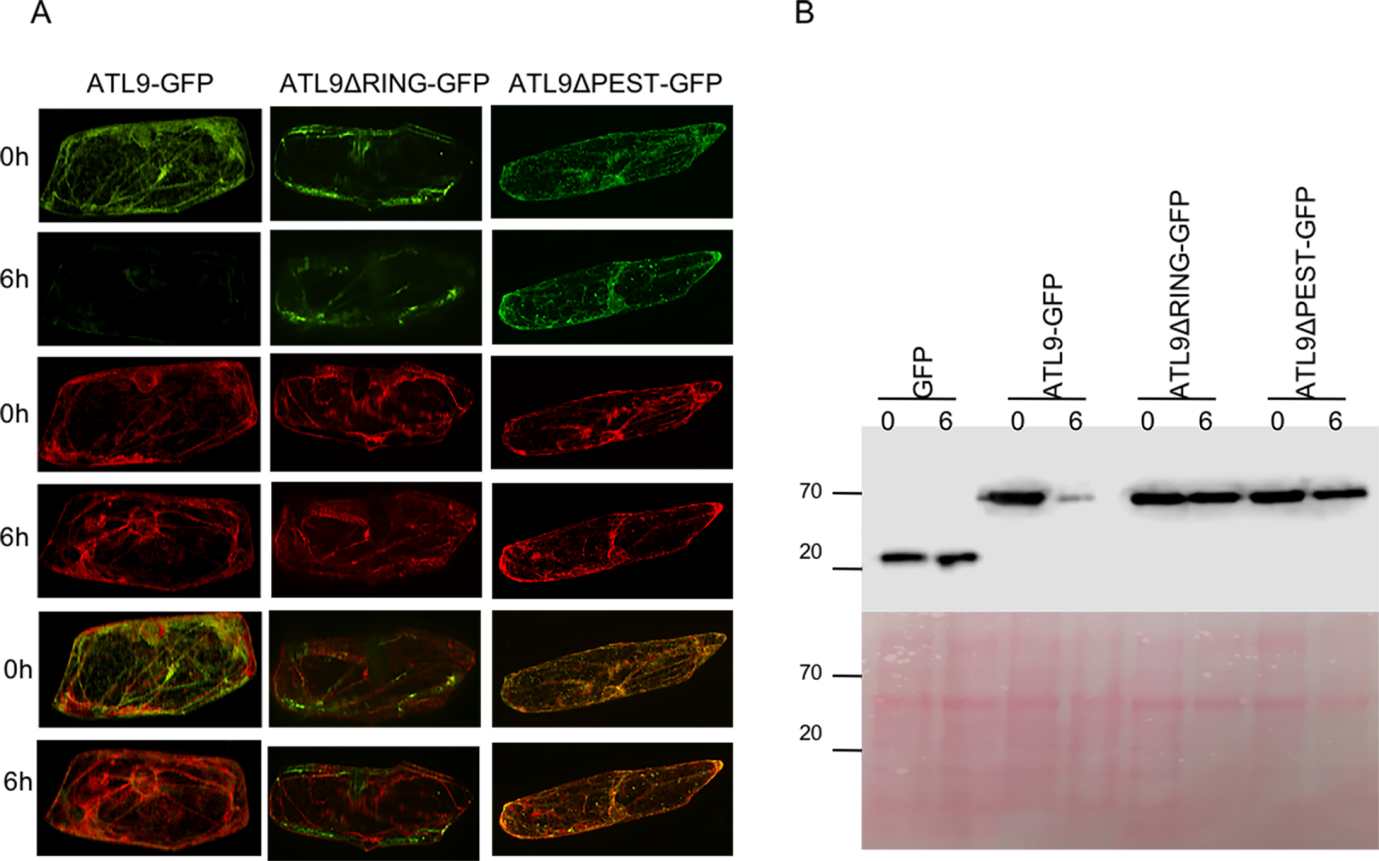
Both the PEST domain and the RING domain contribute to ATL9’s short half-life. (A) Degradation of the ATL9-GFP, ATL9ΔPEST-GFP and ATL9ΔRING-GFP in onion epidermal cells. Method used in this assay is as described in Fig 2. (B) Measurement of ATL9-GFP’s, ATL9ΔPEST-GFP’s and ATL9ΔRING-GFP’s half-life in tobacco leaves by western blot. Method used in this assay is as described in Fig 2. Prior to blocking, the membrane was stained with Ponceau S solution to show equal amounts of protein loaded per lane.

The PEST domain is known to confer a short half-life to proteins degraded via the UPS [31, 32]. Based on this data we hypothesized that the PEST domain of ATL9 might contribute to its short-life span. To examine the function of the PEST domain in ATL9, we constructed a deletion mutant by deleting 20 amino acid residues of the PEST domain (S4A Fig) and used GFP as a reporter (35S:*ATL9ΔPEST-GFP*). The 35S:*ATL9ΔPEST*-*GFP* construct was co-bombarded or co-transformed with the ER-rk marker into onion epidermal cells or *N*. *benthamiana* leaves, respectively. Protein degradation was analyzed using either GFP/mCherry fluorescence ratios or protein immunoblotting as described previously. Both the co-bombardment (Fig 4A) and the protein immunoblotting (Fig 4B) results showed that the ATL9ΔPEST-GFP fusion protein has a longer life span than the full length ATL9-GFP fusion protein. These results correlate significantly with our previous protein degradation experiments, suggesting that PEST domain of ATL9 is essential for its degradation.

ATL9 is a C3-H2-C3 RING zinc finger protein (S4B Fig). It has been shown that the RING domain is essential for the function of ubiquitin E3 ligases [33, 34, 35, 36]. A previous study from our lab showed that ATL9 is a substrate-independent self-ubiquitinating E3 ligase [19]. Our current data show that ATL9 is degraded via a proteasome-dependent pathway. To determine if ATL9 degradation is dependent on its E3 ligase activity, we generated a construct with an inactive RING motif by changing His156 to a Tyrosine residue (35S:*ATL9ΔRING*-*GFP*; S4C Fig). Similar to the experiments with the ATL9ΔPEST-GFP construct, the life span of ATL9*Δ*RING-GFP was observed using both co-bombardment and protein immunoblotting experiments. We found that ATL9ΔRING-GFP showed no detectable loss in protein level after 6 hours compared to ATL9-GFP with an intact RING domain (Fig 4A and 4B). These data suggest that a mutation in the RING domain also confers a longer life-span on the ATL9 protein, lending support to the hypothesis that ATL9’s half-life in *vivo* depends on both its PEST domain and its E3 ligase activity.

### Role of ATL9’s domains in its resistance phenotype

Many studies have demonstrated the importance of cellular localization of immune components for proper disease resistance activation [37, 38, 39, 40, 41, 42]. Our lab previously showed that ATL9 localizes to the ER in plant cells [19]. In this study, we wanted to determine which components of ATL9 were essential for correct protein localization. Using the transmembrane domain deletion mutant of *ATL9*, we found that GFP fluorescence was no longer expressed in the same cellular localization as ATL9-GFP (S4D Fig), suggesting that the loss of the TM domain alters the localization of ATL9 and leads to some loss of function. (S4D Fig).

**Fig 5:**
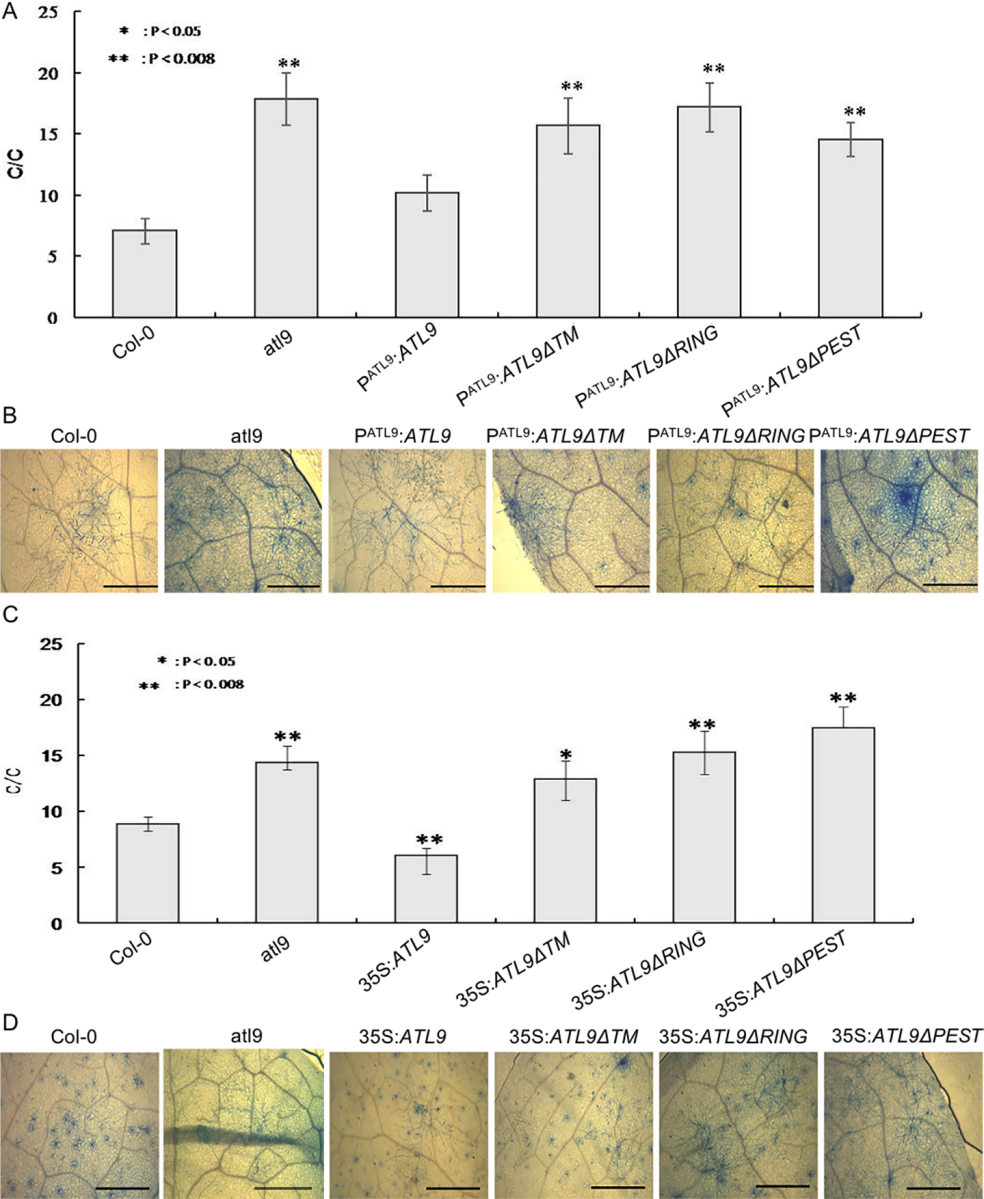
Effect of deletion mutants on resistant phenotype. (A) Inoculation results of P^ATL9^:*ATL9*, P^ATL9^:*ATL9ΔTM*, P^ATL9^:*ATL9ΔPEST* and P^ATL9^:*ATL9ΔRING* transgenic plants. (B) Microscopic disease symptoms of P^ATL9^:*ATL9*, P^ATL9^:*ATL9ΔTM*, P^ATL9^:*ATL9ΔPEST* and P^ATL9^:*ATL9ΔRING* transgenic plants inoculated with *G*. *cichoracearum*. Scale bar is 1mm (C) Inoculation result of 35S:*ATL9*, 35S:*ATL9ΔTM*, 35S:*ATL9ΔPEST* and 35S:*ATL9ΔRING* overexpression plants. (D) Microscopic disease symptoms of 35S:*ATL9*, 35S:*ATL9ΔTM*, 35S:*ATL9ΔPEST* and 35S:*ATL9ΔRING* overexpression transgenic plants inoculated with *G*. *cichoracearum*. Scale bar is 1mm. All transgenic lines used in this experiment are in the *atl9* mutant background. Four-week old *Arabidopsis* were inoculated with powdery mildew and placed in a growth chamber at 22°C day/19°C night with 16 hours of light per 24 hrs. Leaves at 5 dpi were harvested and stained in a trypan blue solution overnight and then decolorized in 95% ethanol overnight. The number of conidiophores per colony was determined from at least 18 leaves taken from 18 plants per genotype. Significance among different samples was analyzed by ANOVA and post hoc test. * indicates p-value < 0.05, when sample compared to Col-0. ** indicates p-value < 0.008 (Bonferroni adjustment p-value), when sample compared to Col-0.

In order to understand the link between the resistance phenotype and correct ATL9 localization, we constructed 35S:*ATL9ΔTM* over-expression transgenic *Arabidopsis* and the P^ATL9^:*ATL9ΔTM* complementary transgenic *Arabidopsis* plants. Furthermore, we also constructed 35S:*ATL9*, 35S:*ATL9ΔPEST*, 35S:*ATL9ΔRING* over-expression transgenic *Arabidopsis* lines and P^ATL9^:*ATL9*, P^ATL9^:*ATL9ΔPEST*, P^ATL9^:*ATL9ΔRING* complementary transgenic *Arabidopsis* to investigate how the RING domain, PEST domain and TM domain affect the resistance phenotype. Homozygous transgenic lines were selected from the T3 generation and subjected to light inoculation with *G*. *cichoracearum*. To assay the resistance phenotype, mature conidiophores were quantified using trypan blue 5 dpi. Our results showed that P^ATL9^:*ATL9* complementary transgenic plants were as resistant as wild-type Col-0 to fungal infection; while P^ATL9^:*ATL9*Δ*TM*, P^ATL9^:*ATL9*Δ*RING* and P^ATL9^:*ATL9*Δ*PEST* complementary transgenic plants were more susceptible to *G*. *cichoracearum*. All deletion mutants showed a similar susceptibility phenotype to the original *atl9* T-DNA insertional mutant (Fig 5A and 5B). Furthermore, inoculation results from 35S:*ATL9*, 35S:*ATL9*Δ*TM*, 35S:*ATL9*Δ*PEST*, 35S:*ATL9*Δ*RING* over-expression transgenic plants also confirmed our conclusion (Fig 5C and 5D). Meanwhile, a transgenic line overexpressing *ATL9* was clearly more resistant to the fungal pathogen than Col-0. These data indicate that increased expression of *ATL9* will enhance the plant’s defense phenotype and that ATL9 function in disease resistance depends on its E3 ligase activity, protein localization, and the PEST domain.

## Discussion

Recent studies have highlighted the importance of the UPS as regulators in the plant resistance response [43, 44, 45, 46, 47, 48, 49, 50]. *ATL* family genes that encode RING-H2 finger proteins are rapidly induced in response to elicitors [51]. Although information on the molecular function of ATL family proteins is limited, several ATL proteins have been found involved in plant innate immunity. EL5, a rice ATL protein, is upregulated by N-acetylchitooligosaccharide and is involved in plant defense responses through the turnover of proteins via the UPS [35]. The *Arabidopsis ATL2* gene has also been shown to be involved in plant defense [51]. In *ATL2* constitutive expression mutants, the expression of the pathogenesis-related genes *NPR1*, *PAL*, *CHS* and *PDF2*.*1* were altered (Serrano and Guzman, 2004). The tobacco *ATL* gene *ACRE-132* is also rapidly induced during both Avr9- and Cf-9*–*mediated defense responses [52]. Previously our group showed that *Arabidopsis ATL9* is involved in both chitin- and NADPH oxidase- mediated defense responses [19]. In this study, we show that *ATL9* is also induced by wounding, suggesting that *ATL9* may be involved in other plant immune responses. Although our knowledge about how plants perceive pathogens and activate associated defense signaling pathways continues to increase, the molecular mechanisms underlying the termination of these responses remain obscure. As excessive or prolonged activation of immune responses are deleterious to the host plant, it is important to understand how plant immunity-related proteins are down regulated. Ubiquitination has been identified as a major player in plant innate immunity attenuation [28]. Two U-box E3 ligases, PUB12 and PUB13 were identified as negative regulators of flagellin signaling by direct ubiquitination of FLS2 [28]. ATL9, which is involved in chitin- and NADPH oxidase- mediated defense responses, is also a self-ubiquitinating E3 ligase. The difference between the GUS activity in P^ATL9^:*GUS* and P^ATL9^:*ATL9-GUS* transgenic *Arabidopsis* suggest that post translational regulation of *ATL9* might be involved. Therefore, understanding the mechanism of its post translational regulation is critical to further reveal the role of E3 ligase in plant defense response. In this study, we found that ATL9 is a short-lived protein and its PEST domain, which is suggested to serve as a proteolytic signal in the UPS, plays an important role in ATL9’s rapid degradation. The RING domain of these proteins has been shown to be essential in numerous studies characterizing E3 ligase activity [53, 54, 55, 56]. In the current study, a His156 to Tyr156 point mutation of ATL9 resulted in prolonged protein stabilization, suggesting that E3 ligase activity also contributes to its degradation. Additionally, the proteasome inhibitor MG132 was shown to prevent the degradation of ATL9, suggesting that ATL9 is degraded via a proteasome-dependent mechanism. Since ATL9 is a self-ubiquitinating E3 ligase [28], we hypothesize that after completing its function in plant immunity, ATL9, as an E3 ligase, ubiquitinates itself by recognizing its own PEST sequences. Then the ubiquitinated ATL9 is transferred to the proteasome where it is degraded [39].

The localization of a resistance protein within the plant cell is essential in mounting an effective disease resistance response. For example, nuclear accumulation of the *Arabidopsis* NB-LRR receptor RPS4, which recognizes the bacterial type III effector AvrRps4, is necessary for triggering immunity [57]. In potato, increasing accumulation of NB-LRR Rx protein in the cytosol leads to enhanced Rx resistance function, while increasing the accumulation of Rx in the nucleus leads to inhibition of Rx resistance functions [37, 39]. The *Arabidopsis* NB-LRR protein RPM1, which recognizes the bacterial effector protein AvrRpm1, is activated at, and functions on, the plasma membrane [15]. Flax L6 protein, which confers resistance to the flax rust phytopathogenic fungus *Melampsora lini*, contains an N-terminal domain that localizes the L6 protein to the Golgi apparatus. Removal of the N-terminal domain reduces L6 resistance function [56], suggesting that the Golgi localization is required for appropriate L6 function. Our group previously showed that ATL9 localizes to ER [19]. In the current work, our data clearly demonstrate that the two transmembrane domains of ATL9 are important for both its localization and its resistance function, suggesting that correct localization of ATL9 is critical for its function.

The presence of a PEST domain is known to expedite protein degradation and previous studies have shown that the PEST domain plays an important role in animal development [53, 54, 55]. In *Drosophila*, phosphorylation of the Cactus PEST domain, which will free Dorsal for nuclear translocation on the ventral and lateral sides of the embryo, is essential for axis formation during development [53]. PEST domain enriched tyrosine phosphatase (PEP) has been identified as an important positive regulator of anaphylaxis in mice [54]. The human NOTCH1 protein plays roles in a variety of developmental processes by controlling cell fate decisions. NOTCH1 PEST domain mutations are an adverse prognostic factor in B-cell chronic lymphocytic leukaemia (B-CLL) [55]. Compared to animals, few studies have tested the role of the PEST domain in plants. In the current study, we show that the PEST domain of ATL9 plays an important role in both protein degradation and its resistance function. To our knowledge, this is the first experimental evidence detailing the importance of PEST domains in plant resistance.

Accumulating evidence strongly suggests that E3 ligases play important roles in plant defense. Previous studies showed that the RING domain is critical for RING-type E3 ubiquitin ligase function [33, 34, 35, 36]. Our data show that both the resistance function and degradation of ATL9 depend on its E3 ligase activity. Since both the PEST domain and RING domain of ATL9 are critical to ATL9 degradation and are important for its resistance function, it is reasonable to hypothesize that post-translational regulation plays an important role in ATL9-mediated plant resistance responses. To further address this question, we are interested in identifying specific targets of ATL9 for degradation via protein-protein interaction. Our previous studies have shown that ATL9 is involved in chitin- and NADPH-oxidase mediated defense, as well as wounding [19], all major defense pathways. Future work will involve identifying the specific targets of ATL9 during the defense response in order to provide more precise information on how ATL9 plays a part in these pathways and assist us in understanding the role of this E3 ligase in overall defense signaling.

## Supporting information

S1 Fig Expression of *CRP1* in *Arabidopsis* tissues at different developmental stages. Different developmental stages of P^CRP1^:*GUS* were subjected to GUS staining at 37°C for at least 4 hours. (A) 2-day old germinating seedling. (B) 4-day old seedling. (C) 7-day old seedling. (D) 3-week old plant. (E) Flower. (F) Siliques. Seedlings and tissues were stained overnight at 37°C in GUS staining buffer. Samples were destained for up to 8 hours in 95% Ethanol and observed. Scale bar: 0.5mm in A-C;1mm in D-F.
(TIF)

S2 Fig *ATL9* is induced by wounding and chitin. (A) P^ATL9^:*GUS* activity in tissue around the wounding treatment. 40-day old transgenic plant leaves was wounded using tweezers, and immediately subjected to GUS staining. We also provided P^ATL2L^:*GUS* as another control for wounding treatment. (B) P^ATL9^:*GUS* activity in leaves with/without chitin treatment 40-day old transgenic plant leaves were treated by 1 mg/mL CSC for 24 hours, and then subject to GUS staining.
(TIF)

S3 Fig Effect of ATL9-GFP and GFP on resistant phenotype. (A) Inoculation result of 35S:*ATL9-GFP* and 35S:*GFP* transgenic plants. (B) Microscopic disease symptoms of 35S:*ATL9-GFP* and 35S:*GFP* inoculated with *G*. *cichoracearum*. Scale bar is 1mm. Transgenic lines used in this experiment are in the *atl9* mutant background. Salicylic acid-deficient NahG transgenic line was served as the negative control. Inoculation method has been described in Fig 5. Significance among different samples was analyzed by ANOVA and post hoc test. * indicates p-value < 0.05, when sample compared to Col-0. ** indicates p-value < 0.008 (Bonferroni adjustment p-value), when sample compared to Col-0.
(TIF)

S4 Fig ATL9 protein structure. (A) Construct of *ATL*9ΔPEST. Amino acids were deleted from wild type sequence. (B) ATL9 protein structure. (C) In the RING motif structure of ATL9 His156 was changed to Tyr156 in the ATL9ΔRING mutant. (D) Subcellular localization of free GFP, 35S:*ATL9-GFP* and 35S:*ATL9ΔTM-GFP*. For the ATL9ΔTM mutant, transmembrane domains were deleted.
(TIF)

S1 Table Data sheet of conidiophores/colony for Fig 5A. Data records and statistical analysis.
(XLSX)

S2 Table Data sheet of conidiophores/colony for Fig 5C. Data records and statistical analysis.
(XLSX)

S3 Table Data sheet of conidiophores/colony for S3A Fig. Data records and statistical analysis.
(XLSX)
