# Supplementary figures and images for: Expression and regulation of ATL9, an E3 ubiquitin ligase involved in plant defense
Source: PLoS One. 2017 Nov 21;12(11):e0188458. doi: 10.1371/journal.pone.0188458 (PMC5697834; doi:10.1371/journal.pone.0188458)

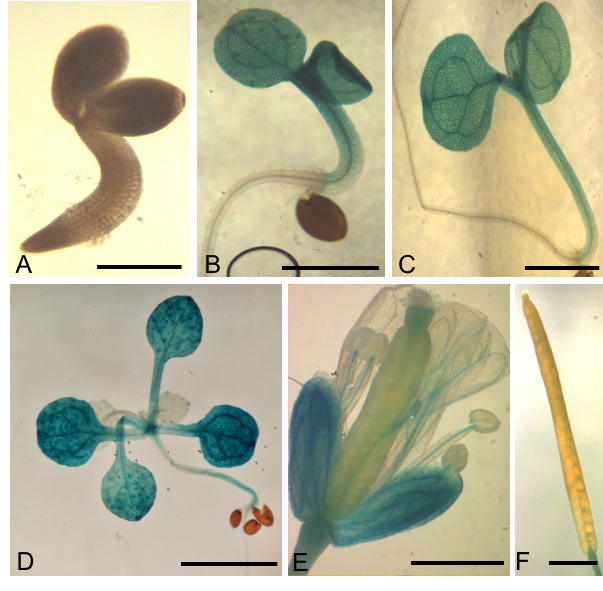

Supplement: S1 Fig — Different developmental stages of PCRP1:GUS were subjected to GUS staining at 37°C for at least 4 hours. (A) 2-day old germinating seedling. (B) 4-day old seedling. (C) 7-day old seedling. (D) 3-week old plant. (E) Flower. (F) Siliques. Seedlings and tissues were stained overnight at 37°C in GUS staining buffer. Samples were destained for up to 8 hours in 95% Ethanol and observed. Scale bar: 0.5mm in A-C;1mm in D-F. (TIF) [file pone.0188458.s001.tif]

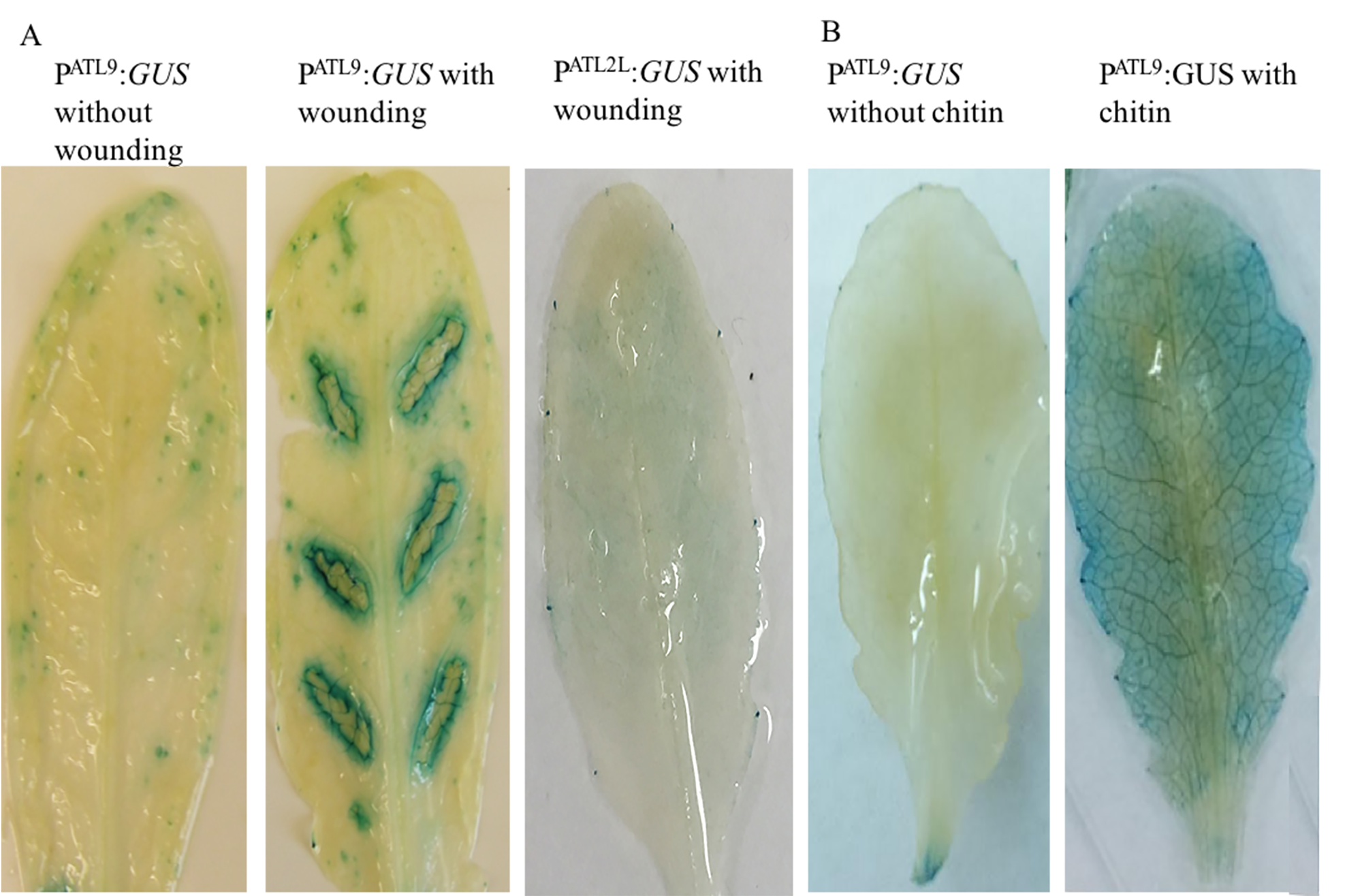

Supplement: S2 Fig — (A) PATL9:GUS activity in tissue around the wounding treatment. 40-day old transgenic plant leaves was wounded using tweezers, and immediately subjected to GUS staining. We also provided PATL2L:GUS as another control for wounding treatment. (B) PATL9:GUS activity in leaves with/without chitin treatment 40-day old transgenic plant leaves were treated by 1 mg/mL CSC for 24 hours, and then subject to GUS staining. (TIF) [file pone.0188458.s002.tif]

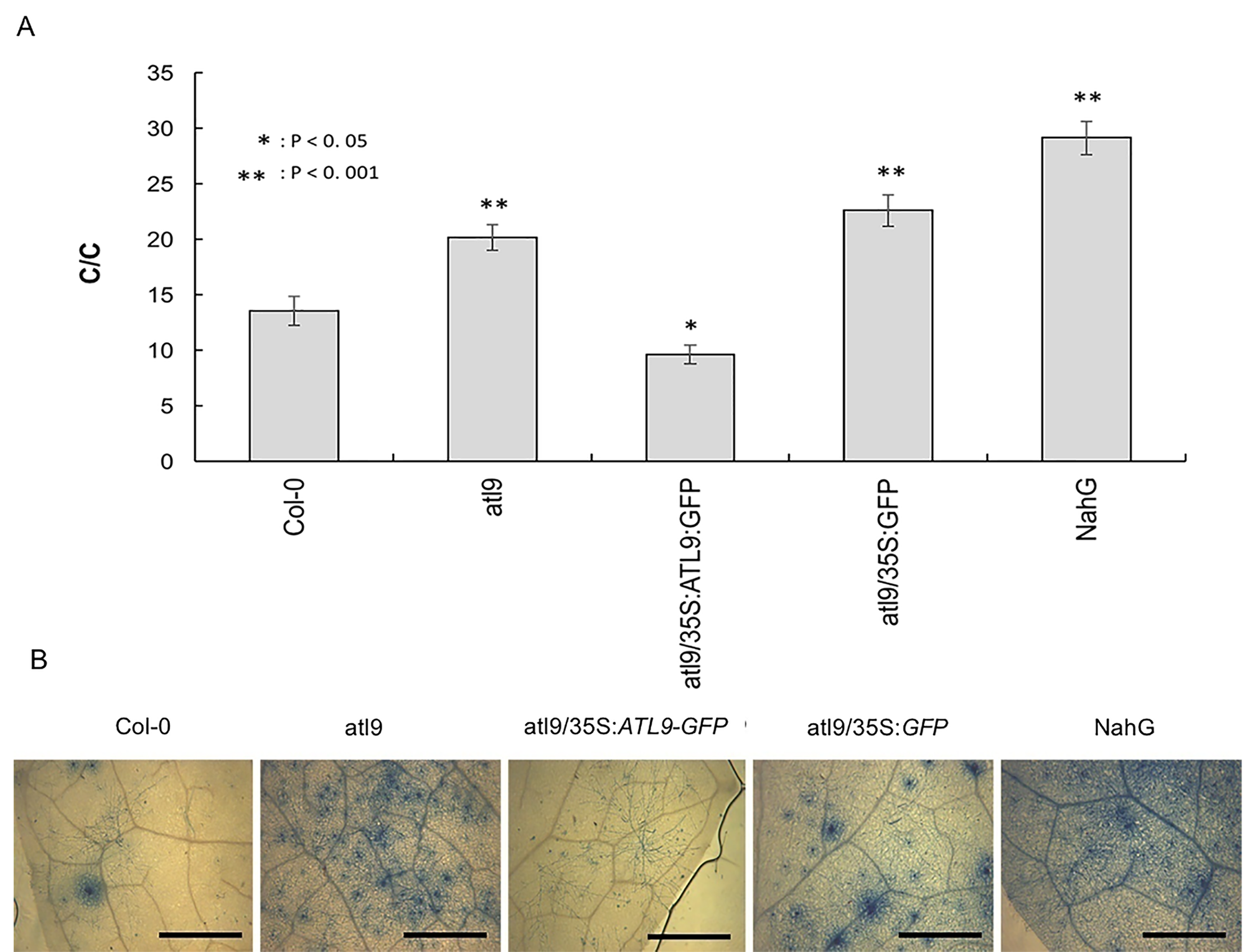

Supplement: S3 Fig — (A) Inoculation result of 35S:ATL9-GFP and 35S:GFP transgenic plants. (B) Microscopic disease symptoms of 35S:ATL9-GFP and 35S:GFP inoculated with G. cichoracearum. Scale bar is 1mm. Transgenic lines used in this experiment are in the atl9 mutant background. Salicylic acid-deficient NahG transgenic line was served as the negative control. Inoculation method has been described in Fig 5. Significance among different samples was analyzed by ANOVA and post hoc test. * indicates p-value < 0.05, when sample compared to Col-0. ** indicates p-value < 0.008 (Bonferroni adjustment p-value), when sample compared to Col-0. (TIF) [file pone.0188458.s003.tif]

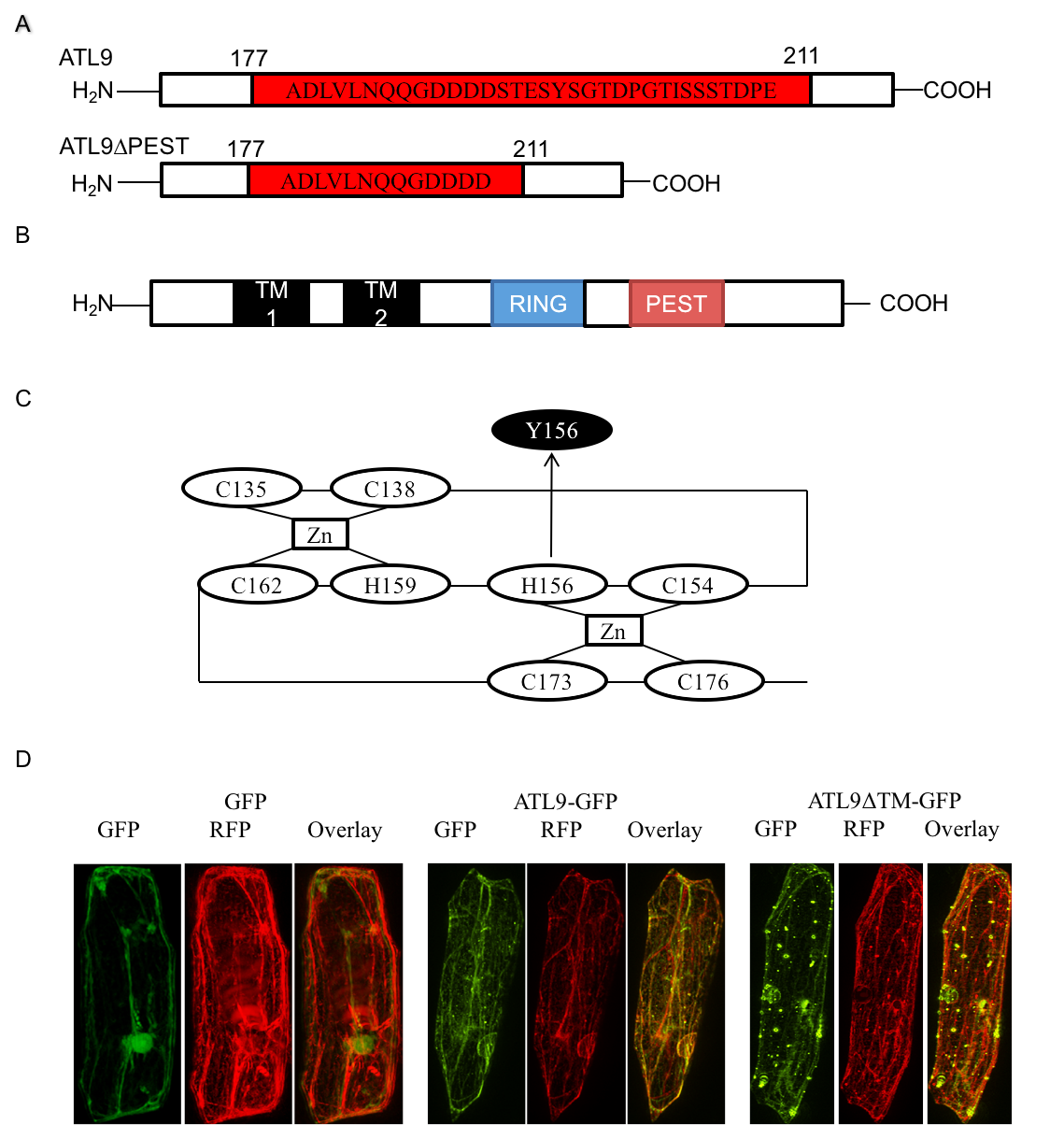

Supplement: S4 Fig — (A) Construct of ATL9ΔPEST. Amino acids were deleted from wild type sequence. (B) ATL9 protein structure. (C) In the RING motif structure of ATL9 His156 was changed to Tyr156 in the ATL9ΔRING mutant. (D) Subcellular localization of free GFP, 35S:ATL9-GFP and 35S:ATL9ΔTM-GFP. For the ATL9ΔTM mutant, transmembrane domains were deleted. (TIF) [file pone.0188458.s004.tif]
